# Uptake and distribution of carboxylated quantum dots in human mesenchymal stem cells: cell growing density matters

**DOI:** 10.1186/s12951-019-0470-6

**Published:** 2019-03-13

**Authors:** Gabrielis Kundrotas, Vitalijus Karabanovas, Marijus Pleckaitis, Marina Juraleviciute, Simona Steponkiene, Zivile Gudleviciene, Ricardas Rotomskis

**Affiliations:** 1 Biobank, National Cancer Institute, Baublio Str. 3b, 08406 Vilnius, Lithuania; 2 Biomedical Physics Laboratory, National Cancer Institute, Baublio Str. 3b, 08406 Vilnius, Lithuania; 3 Department of Chemistry and Bioengineering, Vilnius Gediminas Technical University, Sauletekis Ave. 11, 10223 Vilnius, Lithuania; 4 Laboratory of Immunology, National Cancer Institute, Baublio Str. 3b, 08406 Vilnius, Lithuania; 5 Biophotonics Group of Laser Research Center, Faculty of Physics, Vilnius University, Sauletekis Ave. 9, 10222 Vilnius, Lithuania

**Keywords:** Mesenchymal stem cells, Filopodia-like structures, Extracellular matrix, Labeling, Quantum dots, Fluorescence imaging, Fluorescence-lifetime imaging microscopy

## Abstract

**Background:**

Human mesenchymal stem cells (MSCs) have drawn much attention in the field of regenerative medicine for their immunomodulatory and anti-inflammatory effects. MSCs possess specific tumor-oriented migration and incorporation highlighting the potential for MSCs to be used as an ideal carrier for anticancer agents. Bone marrow is the main source of MSCs for clinical applications. MSCs tracking in vivo is a critical component of the safety and efficacy evaluation of therapeutic cell products; therefore, cells must be labeled with contrast agents to enable visualization of the MSCs migration in vivo. Due to their unique properties, quantum dots (QDs) are emerging as optimal tools in long-term MSC optical imaging applications. The aim of this study was to investigate the uptake dynamics, cytotoxity, subcellular and extracellular distribution of non-targeted carboxylated quantum dots in human bone marrow MSCs at different cell growing densities.

**Results:**

QDs had no negative impact on MSC viability throughout the experiment and accumulated in all observed cells efficiently; however, in some MSCs QDs induced formation of lipid droplets. At low cell growing densities QDs distribute within MSCs cytoplasm already after 1 h of incubation reaching saturation after 6 h. After 24 h QDs localize mainly in the perinuclear region of the cells in endosomes. Interestingly, in more confluent culture QDs localize mostly outside MSCs. QDs abundantly mark MSC long filopodia-like structures attaching neighboring cells. At high cell density cultivation, we for the first time demonstrated that carboxylated QDs localize in human bone marrow MSC extracellular matrix. Moreover, we observed that average photoluminescence lifetime of QDs distributed in extracellular matrix are longer than lifetimes of QDs entrapped in endocytic vesicles; thus, for the first time showing the possibility to identify and distinguish localization of QDs in various extracellular and intracellular structures using fluorescence-lifetime imaging microscopy without additional staining assays.

**Conclusion:**

Carboxylated QDs can be used as nonspecific and effective dye for staining of human bone marrow MSCs and their specific extracellular structures. These results are promising in fundamental stem cell biology as well as in cellular therapy, anticancer drug delivery and tissue engineering.

**Electronic supplementary material:**

The online version of this article (10.1186/s12951-019-0470-6) contains supplementary material, which is available to authorized users.

## Background

Human mesenchymal stem cells (MSCs) are a diverse subset of fibroblast-like precursor cells with high self-renewal capacity present in the stromal fraction of many adult tissues [1]. During the last decade MSCs have drawn much attention from basic and translational investigators in the field of regenerative medicine, mainly due to their multipotent differentiation capacity, immunomodulatory and anti-inflammatory effects [2]. Though the nature and functions of MSCs remain not fully clear, several clinical trials have underscored their effectiveness in treating different illnesses, including hematological, inflammatory, cardiovascular, bone and cartilage, neurological and autoimmune diseases [3].

MSCs are particularly promising in cancer therapy. The specific tumor and their metastases-oriented migration and incorporation of MSCs have been demonstrated in various pre-clinical models, highlighting the possibility of modifying these cells to express anticancer molecules and using them as an ideal carrier for anticancer agent delivery [4]. The role of MSC as carriers of drug delivery systems offers an alternative therapeutic approach capable of overcoming clinical restrictions related to the systemic administration of antitumor agents including cytokines, interferons or pro-drugs with short half-life and high toxicity [5]. Recently, the use of modified MSCs as therapy vehicles for the treatment of solid tumors has progressed to the first generation of clinical trials [6].

Cell tracking is a critical component of the safety, efficacy and mechanism of action evaluation of therapeutic cell products [7]. To track MSCs, cells must be labeled with a contrast agent prior to transplantation to make them visible within the body [8]. Therefore, fluorescent imaging [9] and magnetic resonance imaging (MRI) [10] technologies based on nanoparticles have been developed to monitor MSC after injection. Nanotechnology-based cell-tracking methods provide non-toxic, non-invasive, clinically applicable solutions for long-term monitoring of cells post-injection [11]. Superparamagnetic iron oxide (SPIO) nanoparticle based MRI is among the most widely employed for in vivo monitoring of the stem cells [12, 13]. However, limited image resolution makes it challenging to accurately detect small numbers of cells after transplantation with MRI [14]. Also, MRI may overestimate the true size of the MSC grafts [15]. Moreover, SPIO-based MRI may not be suitable for long-term tracking of transplanted MSCs in vivo compared to MSCs labeled with fluorescent protein [16].

The most popular labelling markers for fluorescence imaging are organic dyes. However, most organic dyes can only be used for short-term imaging because of photobleaching effect [17]. Another emerging type of fluorescent labels are quantum dots (QDs) which are fluorescent semiconductor nanoparticles with unique optical and chemical features which make them useful as fluorescent tags for long-term in vitro and in vivo cell imaging applications. QDs have narrow band emission spectra and broad excitation spectra and are resistant to chemical and metabolic degradation [18]. QDs possess improved signal brightness as well as enhanced resistance to photobleaching compared to conventional organic and protein fluorophores [19].

Using QDs for the labeling and tracking purposes of human mesenchymal stem cells have been firstly demonstrated in the study of Hsieh et al. [20]. Furthermore, QDs were specifically modified with biologically active molecules making the imaging of particular MSCs structures possible [21]. Another potential application of bioconjugated QDs is effective regulation of MSCs differentiation [22], which is important for understanding of MSCs behaviors in vitro and in vivo [23]. Recently, we have shown that non-targeted carboxyl-coated QDs are biocompatible with human skin MSCs, not affecting cell viability, proliferation, immunophenotype and ability to differentiate [24]. Moreover, in 3D spheroid co-culture and human tumor xenograft model we have demonstrated that MSCs nanoengineered with QDs could serve as a vehicle for targeted drug delivery to metastatic cancer [25, 26]. These results are in compliance with observations of other groups. Ohyabu et al. showed that QDs are efficient, genetically noninvasive, nontoxic, and functionally inert way to label human MSCs [27]. Tao et al. demonstrated that human bone marrow MSCs labeled with QDs remain viable on biological sutures transplanted in vivo [28]. Based on these observations, we hypothesize that carboxylated QDs would be an optimal tool for human MSC labeling and imaging. Bone marrow derived MSCs are the most frequently investigated cell type and often designated as the gold standard [29]. However, significant number of MSCs is needed for effective cell mediated therapy [30], thus it is necessary to label large amount of MSCs with QDs simultaneously.

In this study, we for the first time investigated the distribution of non-targeted carboxylated quantum dots with emission peak at 625 nm in human bone marrow MSC culture at different cell growing density. Surprisingly, at 20,000 cells/cm^2^ cell growing density, these nanoparticles localized not only inside the MSCs, but also on cell protrusions, as well as irregularly outside the cells highlighting extracellular matrix.

## Results

### QDs intracellular uptake dynamics and biological effects on MSCs

**Fig. 1:**
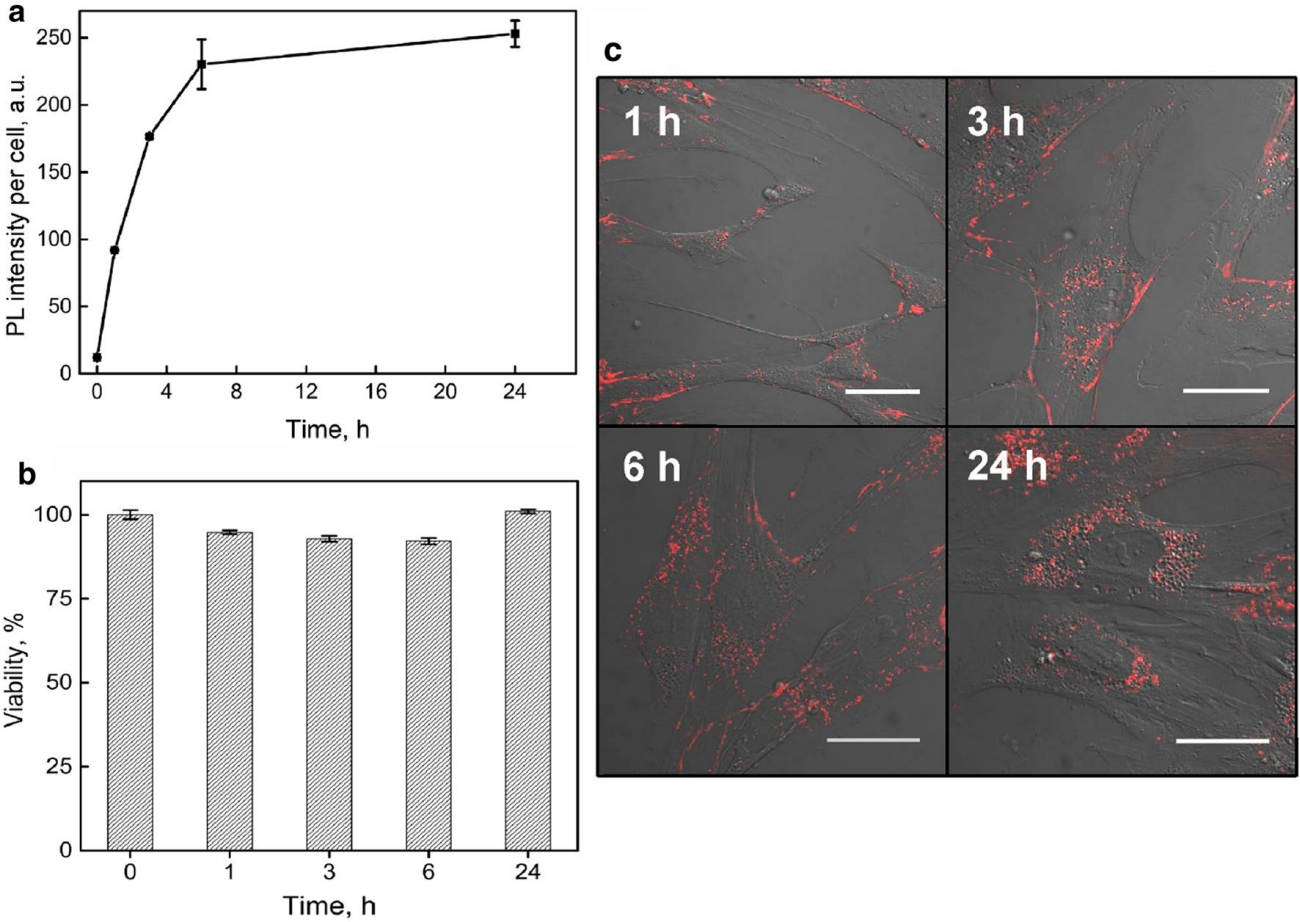
Carboxylated quantum dots intracellular uptake dynamics and biological effects on MSCs. **a** Dynamics of accumulation for QDs in MSCs. **b** MSCs viability at different incubation times with QDs. **c** MSCs morphology and intracellular distribution of QDs after various times of incubation. Red color exhibits QDs distribution in MSCs culture

After isolation from bone marrow, MSCs for characterization were expanded in vitro until passage 3. Before QDs labeling experiments, identity of MSCs were confirmed by adherence to plastic, morphology, immunophenotype, proliferation capacity and genomic stability. Results of MSC analysis we have published previously [31], showing long spindle-shaped or flat fibroblast like MSC morphology with 99% of the cells stained positive for surface markers CD44, CD73, CD90 and CD105 and more than 98% of the cells stained negative for antigens CD11b, CD19, CD45, CD34 and HLA-DR. Viability of cultivated and non-treated MSCs varied between 87.0 and 96.6% in this study (data not shown). To evaluate the temporal accumulation dynamics of QDs into MSCs at 5000 cells/cm^2^ seeding density, the photoluminescence (PL) intensity of QDs at 625 nm was registered by the means of flow cytometry until 24 h of incubation. The intracellular QDs photoluminescence intensity signal gradually increased and started to saturate after 6 h of incubation. Saturation remained throughout the rest time of the experiment (Fig. 1a).

The time dependent cytotoxicity at QDs concentration of 8 nM was assessed. Incubation of MSCs with QDs did not change cell viability significantly during the accumulation experiments (accumulation time 1–24 h) and varied within 8% error limits (Fig. 1b). It should be stressed that labeling with QDs did not change MSCs morphology and no obvious features of apoptosis (cell rounding, cell and nuclei fragmentation) were observed (Fig. 1c).

### QDs localization in MSC culture

**Fig. 2:**
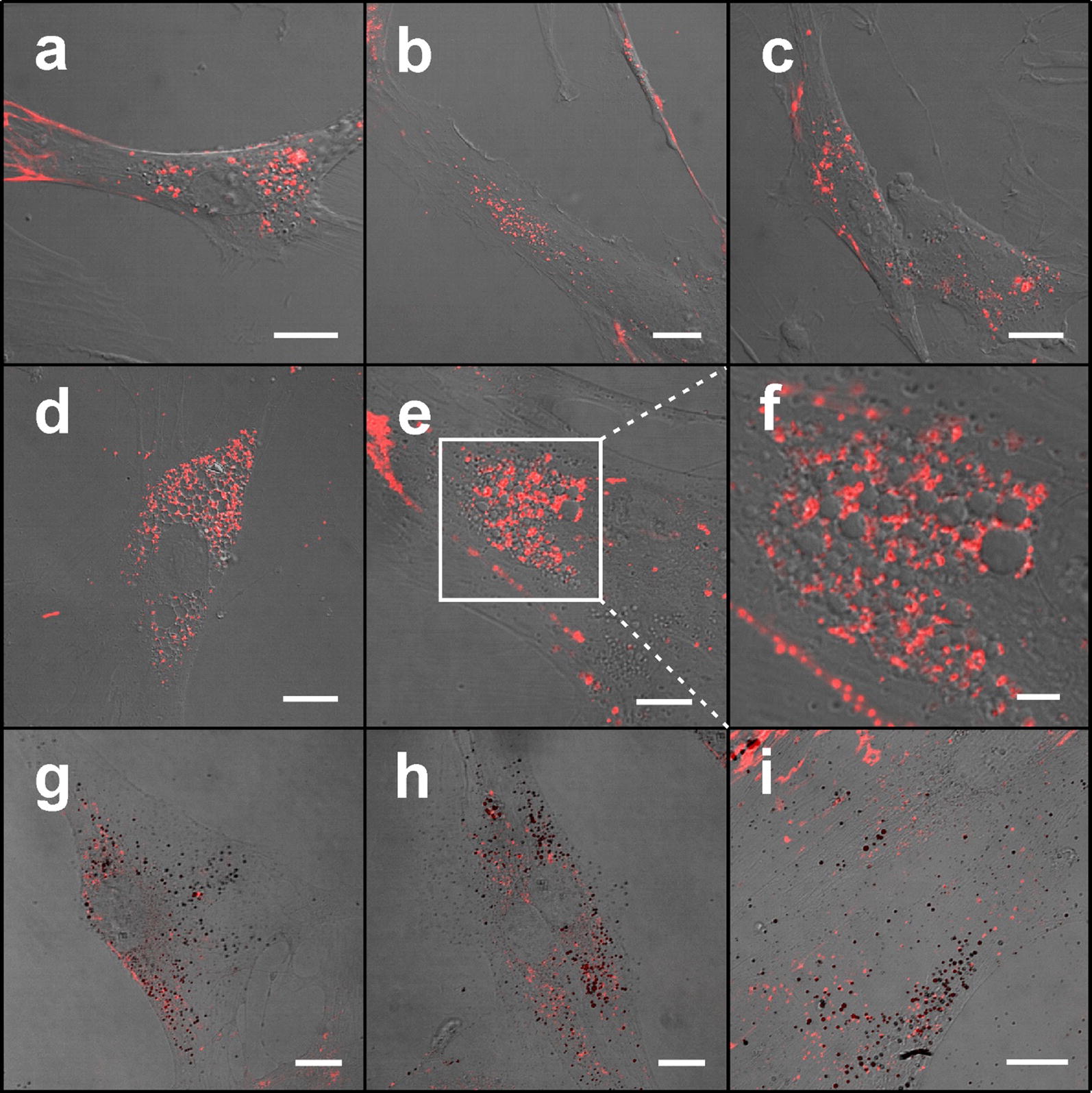
Distribution of carboxylated quantum dots in MSCs at different incubation times. Confocal fluorescence microscopy images showing the distribution of QDs in MSCs after 1 h (**a**), 3 h (**b**), 6 h (**c**), 24 h (**d**–**f**) incubation. Enlarged part of MSC containing hollow vesicles surrounded by smaller QDs-fulfilled vesicle-type structures after 24 h incubation (**f**) shown with white rectangle in **e**. MSCs incubated with QDs and additionally stained with Oil Red O dye show adipogenic-like phenotype of MSCs 24 h after treatment with QDs (**g**–**i**). Scale bars 15 µm (**a**–**e**, **g**–**i**) and 5 µm (**f**)

**Fig. 3:**
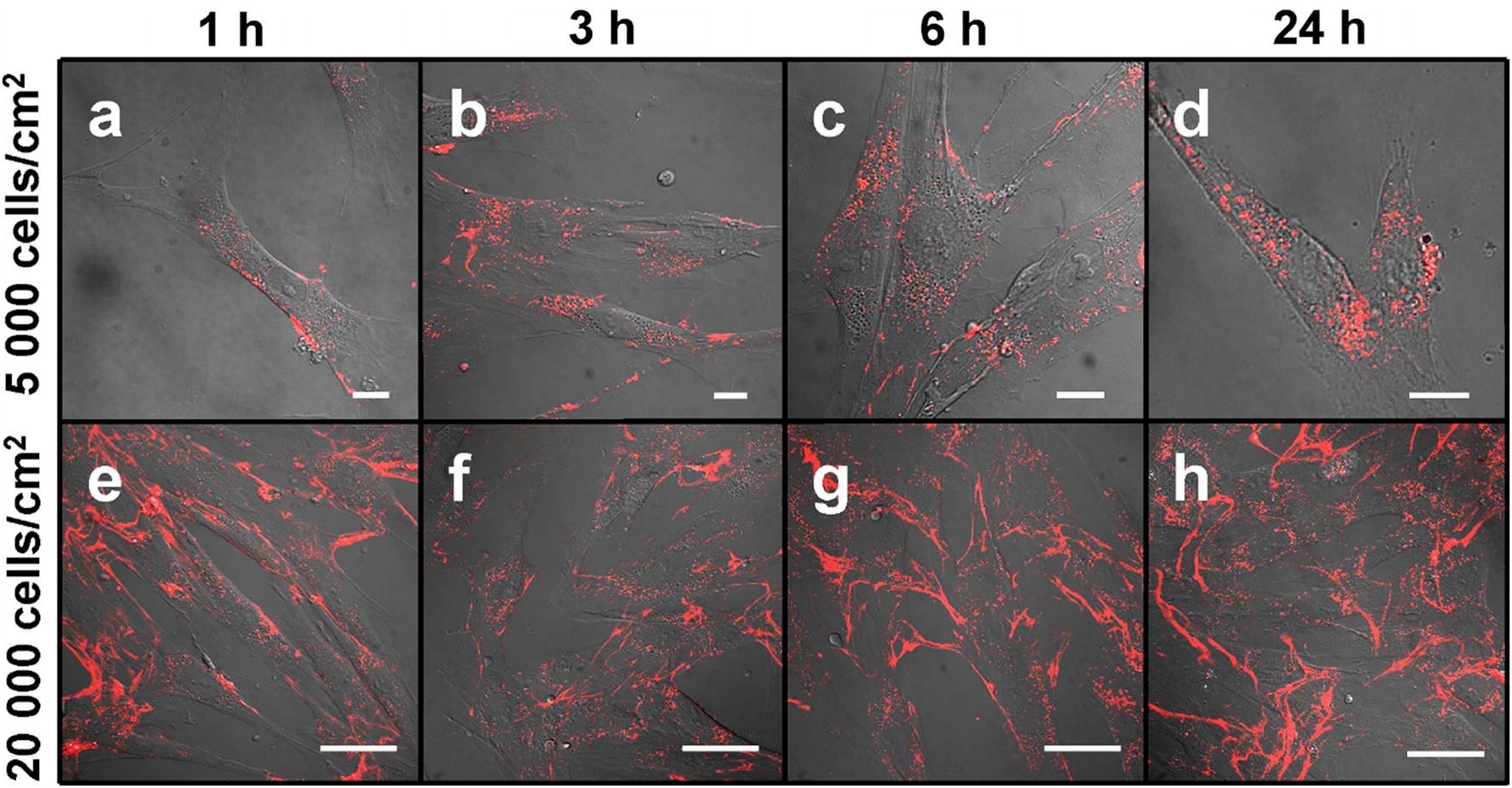
Confocal fluorescence microscopy images showing carboxylated quantum dots localization in MSC culture at different cell cultivation densities. At low cultivation density (5000 cells/cm^2^; top row), QDs (red) distribute intracellularly and at high cultivation density (20,000 cells/cm^2^; bottom row), QDs deposit on long cytoplasmic processes attaching cells 1 h (**a**, **e**), 3 h (**b**, **f**), 6 h (**c**, **g**) and 24 h (**d**, **h**), respectively, after QDs introduction. Scale bars 15 μm (**a**–**d**) and 50 μm (**e**–**h**)

Accumulation of QDs in MSC after 1, 3, 6 and 24 h of incubation was investigated. Three different types of images of accumulation of QDs [inside MSCs (Fig. 2), on MSC particular surface parts (Fig. 3) and outside the MSCs (Fig. 5)] dependent on seeded cells density were detected.

Vesicle-type structures (endosomes) filled with QDs and spread throughout the cytoplasm already 1 h after introduction were detected (Fig. 2a). The number and size of these vesicles inside the cells increases with incubation time together with the increasing of the intensity of PL of QDs (Fig. 2). After 24 h treatment in some cells we observed large vesicles positive for Oil Red O staining (Fig. 2g–i) without nanoparticles, clustered around the nucleus and surrounded with endosomes filled with QDs (Fig. 2e, f).

Confocal fluorescence microscopy analysis showed that in more confluent MSC culture seeded at 20,000 cells/cm^2^ QDs localize not only inside the cells, but attach mainly on some surface structures of the main cell body and on the long filopodia-like structures which contact neighboring cells, as well as on the complex extracellular structures which are not characteristic for single MSCs (Fig. 3a–d) and which are oriented not in the same directions as the cells themselves (Fig. 3e–h). Also see Additional files 1 and 2.

After cell harvesting from flask surface, we detected that QDs remain attached to extracellular structures and the use of trypsin does not affect their properties (data not shown). The differential interference contrast (DIC) microscopy was applied to observe these structures, because extracellular structures of non-labeled (with QDs) human MSCs were not detectable with conventional bright-field microcopy.

**Fig. 4:**
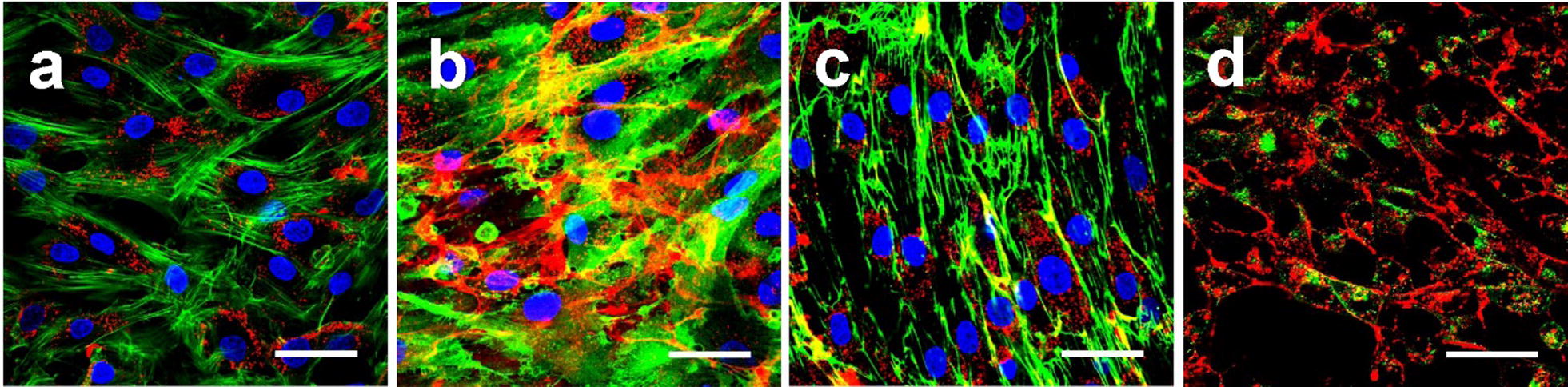
Staining of MSC intracellular and extracellular molecules. MSCs labelled with actin staining Alexa-Fluor 488 nm-conjugated phalloidin (green, **a**), mouse anti-human CD44 antibody conjugated with Alexa-Fluor 488 (green, **b**), Gibco fibronectin (green, **c**) and Alexa-Fluor 488 nm-conjugated transferrin (green, **d**) after 24 h of incubation with QDs (red). Scale bars for all images 50 μm

After 24 h incubation, confocal immunofluorescence microscopy images demonstrated that quantum dots extracellularly co-lozalize with the CD44 and fibronectin, and intracellularly with the transferrin (Fig. 4b–d).

**Fig. 5:**
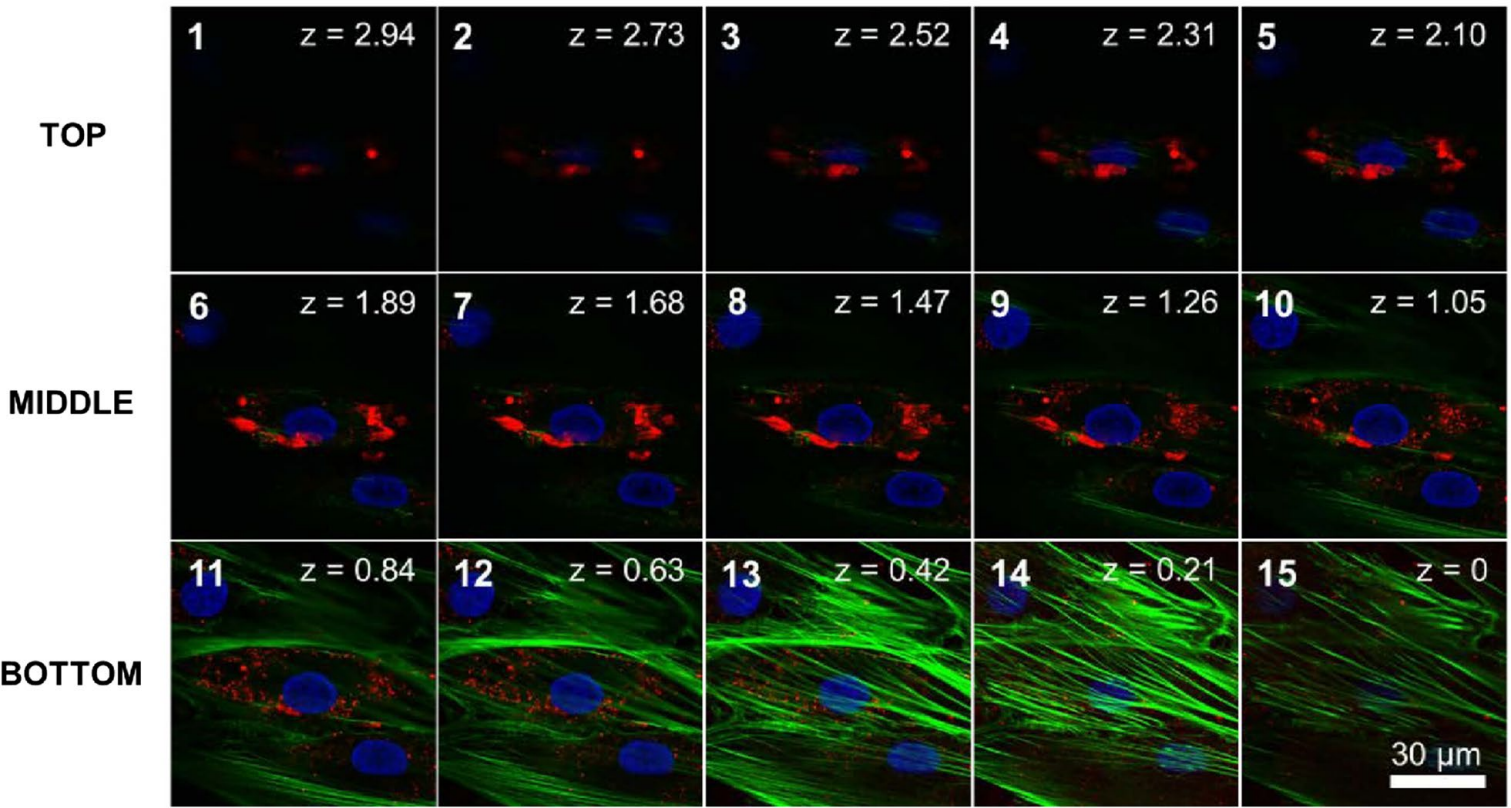
Z-stack imaging series of MSCs treated with carboxylated QDs for 24 h. Z-stack images were collected at 0.2 µm steps by confocal laser scanning microscopy. Green corresponds to Alexa-Fluor 488 nm-conjugated phalloidin labeling actin, red corresponds to QDs and blue corresponds to Hoechst labeling nucleus. Scale bar for all images 30 µm

Confocal laser scanning microscopy analysis identified QDs on the middle and the upper scanning layers of the picture in Z dimension localizing above MSC intercellular actin and nucleus (Fig. 5, Additional files 3 and 4).

**Fig. 6:**
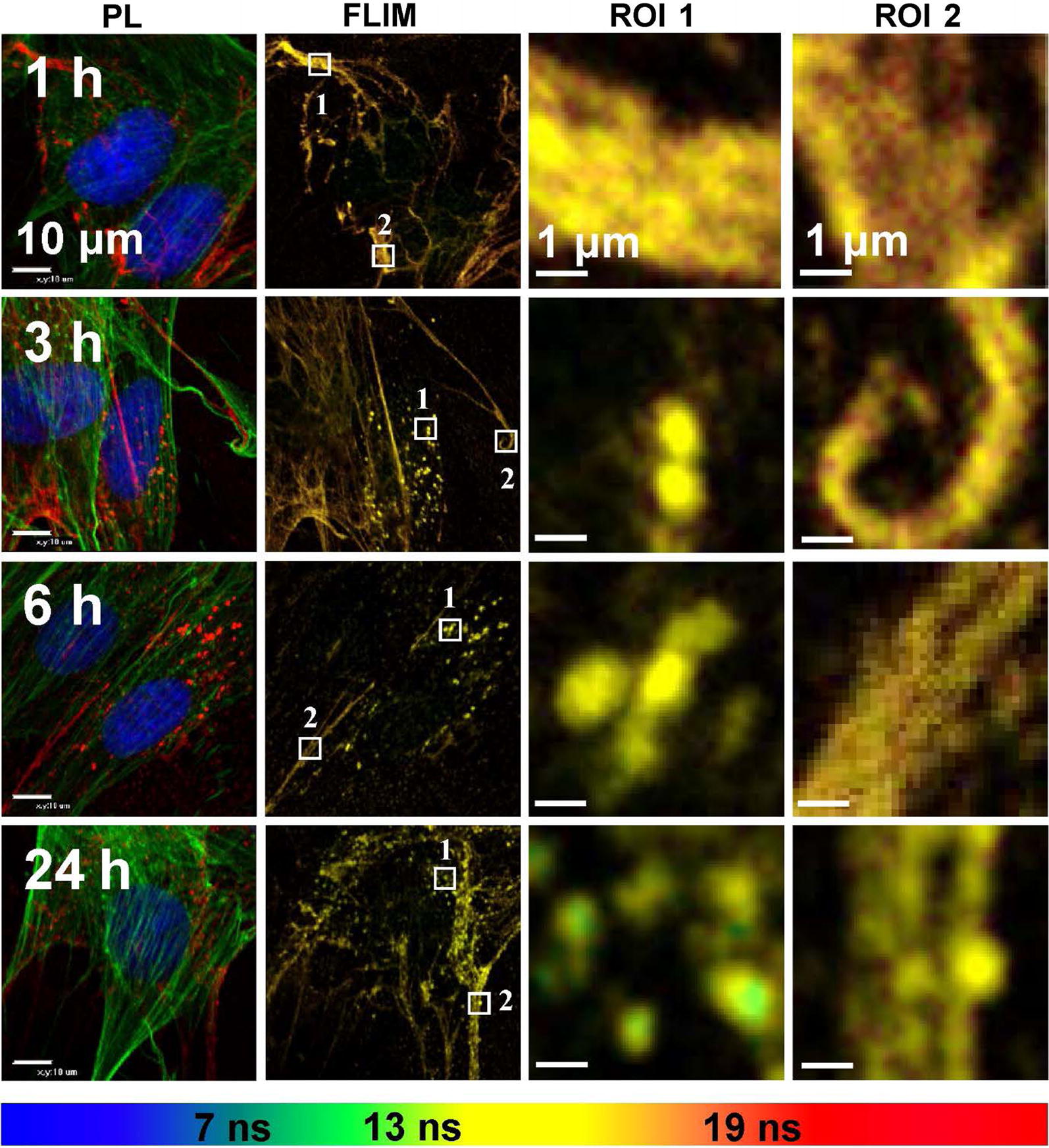
Confocal photoluminescence images of MSCs at different times of incubation with carboxylated QDs showing intracellular distribution. FLIM images of MSCs with marked regions of interest (ROI) which are enlarged in columns ROI 1 and ROI 2. Scale bars for PL and FLIM images 10 µm and 1 µm for enlarged ROI. FLIM images are overlaid and in different lifetime decay gates (< 7 ns—blue, 7–13 ns green, 13–19 ns yellow and 19–29 ns—red)

Fluorescence lifetime imaging microscopy (FLIM) images were registered at four defined time gates that allowed us to study the spatial localization of QDs (Fig. 6). The presence of time-related differences in the mean PL lifetime intracellular distribution implies that the intravesicular QDs are surrounded with different microenvironments [32, 33]. In contrast, if the QDs surrounding media were homogenous, the FLIM images would be identical and only the PL intensity would differ. Two regions of interest (ROI) were inspected from each FLIM image at various incubation times which enabled the identification of how the different phases in uptake of QDs affects PL of these nanoparticles. The results showed that PL lifetimes of QDs accumulated in extracellular matrix or membrane were longer than lifetimes of QDs in endocytic vesicles. PL decayed even more during maturation of endolysosomal structures [33]. In addition, the results revealed the tendency for PL lifetimes to become shorter the longer time of incubation with QDs is which supports the idea of spatial heterogeneity of intracellular vesicles filled with carboxyl-coated QDs. Mean PL lifetimes of QDs distributed on the extracellular matrix were from ~ 18.5 ns after 1 h of incubation decaying to ~ 17.1 ns after 24 h. For QDs accumulated on membrane of MSCs mean PL lifetime 1 h after introduction of QDs also was ~ 18.5 ns. However, it decayed less during time and after 24 h mean lifetime of QDs was about 17.8 ns. Finally, FLIM allowed distinguishing of QDs accumulated intracellularly. PL decayed from ~ 17.5 to ~ 14.3 ns after 1 h and 24 h of incubation with QDs, respectively.

## Discussion

Previously we have shown that human bone marrow MSCs isolated from patients by a novel donor-friendly methodology [34] retain their genomic stability and stemness characteristics in early culture passages [31]. We confirmed MSCs identification and homogeneity by adhesion, morphological and flow cytometry [31] criteria as recommended by the International Society for Cellular Therapy [35]. Real-time in vivo cell tracking can be performed by labeling cells with probes that enter the cell by active or passive transport and are trapped intracellularly [36]. Despite the increasing popularity of QDs as cell labeling agents, their potential cytotoxicity remains a major issue among academic, industrial, and regulatory communities [37].

As it is seen from our experiments, the human bone marrow stem cells accumulate the QDs and do not exhibit toxicity after 24 h of incubation at concentration 8 nM. We did not detect any increase of debris or dead cells (Fig. 1c) showing no cytotoxic effect of QDs on the cells. MSC viability was considerably higher than 80% throughout the experiment (Fig. 1b); therefore, QDs can be recognized as biocompatible [38]. We consider the experiment time of 24 h to be sufficient because MSCs are expected to be transplanted within 24 h of QD labeling [39]. To sum up, here we show that human MSCs are efficiently labeled with QDs with no cytotoxity.

We also showed that human bone marrow MSCs uptake QDs efficiently. Cellular uptake of QDs increases during the first hours of incubation and reaches plateau within 6 h (Fig. 1a). These results are consistent with QDs uptake dynamics in human skin MSCs [26]. Several reasons could lead to the saturation. MSCs could stop up-taking more QDs due to internal biochemical mechanisms (for example, due to no free membrane endocytosis receptors) or because of exhaustion of QDs in the medium.

Moreover, specific accumulation of QDs were detected in stem cell in comparison with other cell type cultures [40]. The extraordinary extracellular and intracellular distribution of QDs were detected (Fig. 3) whereas in other cell type cultures QDs distribute in vesicles and distributed uniformly in the cell under prolonged incubation [40]. Accumulated in MSCs culture QDs localize at three different levels (inside MSCs, on particular MSC surface parts and outside the MSCs) dependent on seeded cells density. At each level QDs localize irregularly and thus highlight special structures of in vitro cultivated human bone marrow MSCs. In addition, here we confirmed high expression of CD44 (Fig. 4b) which is specific marker of stemness and mesenchymal lineage [41].

QDs label MSCs specifically because we did not determine QDs in the background throughout the experiment. Practically, all MSCs displayed red fluorescence signals showing that all the cells were labeled with QDs. Intracellular QDs are already detectable at 1 h of incubation. The brightness of signals increased until 6 h and remained such until 24 h (Fig. 1c). These fluorescence confocal microscopy results are in line with flow cytometry results (Fig. 1a). We noticed that QDs accumulate in human bone marrow MSCs spontaneously similar like in mouse fibroblasts which uptake QDs by natural endocytosis [32] and distribute throughout the cytosol (Fig. 2). However, we observed the differences already at the first time point of observation. Vesicles filled with QDs distribute within MSCs already 1 h after introduction and no QDs were detected on plasma membrane (Fig. 2a). Our results correlate with results of other teams showing that QDs with emissions at 520 nm, 525 nm, 605 nm and 645 nm firstly disperse throughout the cytoplasm, then after 24 h localize mainly in the perinuclear area of bone marrow MSCs in endosome-like structures [20, 42, 43]. Intracellular co-localization of QDs with transferrin after 24 h shows that quantum dots accumulate in MSC by clathrin-dependent endocytosis pathway [32] and afterwards fuse with transferrin containing endosomes (Fig. 4d). The presence of endosomes on actin filaments demonstrates the transportation of these vesicles, but also implies that once inside the endosomes, QDs cannot escape and stain intracellular structures (Fig. 4a). Cumulatively, these results demonstrate that human MSCs are efficiently labeled with QDs and suggest 6 h of MSCs incubation with QDs to be optimal.

Moreover, we found that in some vesicles clustered around the nucleus QDs do not accumulate and these were surrounded with smaller vesicles filled with QDs. It is possible that these two types of vesicles do not fuse because we haven’t detected their fusion throughout the experiment. After staining with Oil Red O some of these QD-negative vesicles were seen as bright red spheres, a characteristic of human MSC adipocytic phenotype [44]. Lipid droplets consist of apolar lipids and other hydrophobic substances and are surrounded by amphiphilic proteins [45]. QDs have attached hydrophilic carboxyl groups and that could explain why these vesicles do not fuse. However, in our study MSCs did not change in morphology from the fibroblastic shape into and adipocyte-like spherical shape what is typical for adipocytes derived from human MSCs [46]. Lipid droplets were more prominent in 24 h treated MSCs (Fig. 2g–i), leading to conclusion that these structures are QDs induced. Saulite et al. [24] showed that carboxyl-coated QD655 do not induce human skin MSC spontaneous differentiation and do not alter ability to differentiate into adipocytes. Meanwhile, it was demonstrated that silver nanoparticles enhance human bone marrow MSC adipogenesis [47] and graphene QDs enhance adipogenic differentiation of rat bone marrow MSCs [48]. We presume that adipogenic features observed in this study may reflect some degree of the intrinsic bone marrow MSC adipocytic commitment [49] that might have been promoted by QDs.

Our next series of experiments were focused on QDs localization on MSC surface and extracellular structures that are obviously seen in fluorescence imaging at 20,000 cells/cm^2^ MSC culture density (Fig. 3e–h). The localization of the QDs on the surface of subconfluencial MSCs was exclusive. QDs co-localized with cell surface marker CD44 (Fig. 4b), confirming QDs position on MSC outer membrane. It should be noted that QDs concentrated mainly on the long cell membrane protrusions of connected adherent cells (Fig. 3e–h). Such layout of QDs is not characteristic for other cell types, such as fibroblasts or cancer cell lines [40], indicating specificity of MSC projections. Sectioning with a confocal laser scanning microscope showed that QDs localize on the middle and upper scanning layers lacking MSC cellular actin, thus indicating the extracellular position (Fig. 5). It was confirmed by revealing QDs co-localization with fibronectin (Fig. 4c) which is essential and ubiquitous extracellular matrix (ECM) glycoprotein [50]. These observations were further confirmed by showing QDs localization with CD44 (Fig. 4b) which has been described as the main receptor that modulates cell-ECM interactions [51]. Chen et al. demonstrated that CD44 molecules on human umbilical cord MSCs are predominantly located on the peak of the membrane protrusions, which may enhance CD44 binding properties to the ECM [52]. CD44 mediate cell adhesion to and migration through the ECM, and Ke et al. showed that CD44 is involved in migration of human umbilical cord MSCs [53]. Cumulatively, these results strongly point out that these complex structures are likely to be the ECM. In this study, for the first time to our knowledge we demonstrate the MSC-deposited ECM by labeling it with non-targeted carboxylated quantum dots. QDs are negatively charged; therefore, it is likely that QDs interact nonspecifically with positive sites of ECM, thus revealing its full picture.

ECM is an important component of the cellular microenvironment, supplying critical biochemical and physical signals to initiate and sustain cellular functions [54]. It is known that MSCs in vitro alter their niche to allow cell attachment by synthesizing their own ECM proteins. Several studies have shown that human bone marrow MSCs deposit ECM in vitro [55, 56]. It might at least partially explain the QD-negative vesicles we detected in the perinuclear region (Fig. 2d) which are associated with intense protein synthesis [57]. We noticed that ECM is detectable only in cell culture with higher confluence (Fig. 3e–h). It is in compliance with literature data showing that confluent human MSCs preferentially express genes for ECM proteins when comparing with pre-confluent culture [58]. We also noted that the denser ECM is, the lesser QDs are inside the cells (Fig. 3). Transferrin still enters MSCs and partially co-localizes with QDs (Fig. 4d); therefore, ECM is not likely to act as a barrier for QDs to get to the cell membrane. All these findings indicate that QDs could be secreted together with ECM proteins.

FLIM gives an efficient and unique approach to study cells and their interactions with nanoparticles because of its versatility, specificity and relatively high sensitivity. By analyzing the characteristics of PL, it becomes possible to visualize and study complex dynamic events and interactions of nanoparticles with surrounding environment in cells, organelles and sub-organelle components within the biological specimen [59, 60]. PL spectra of QDs, localized inside or outside the cells, usually can be similar or hardly distinguishable. Thus, FLIM allows making a distinction between different phases of incubation and accumulation of QDs. In previous our study, we showed that QDs accumulation is not only time-dependent but also depends on localization inside/outside the cells. Moreover, the changes in mean PL lifetime of QDs registered during their accumulation most likely can be addressed to the variations in biomolecular composition and acidity of the microenvironment altering the molecular interactions with QDs, which can affect PL properties of QDs [33]. These findings support the results of our current study, in which FLIM analysis results show that QDs located outside MSCs have longer lifetimes than QDs located inside the cells (Fig. 6). We denoted the advantages of applying FLIM for effectively studying time-dependent accumulation and dynamics of QDs within MSCs both intracellularly and extracellularly. Even more, MSCs labeling technology with QDs can be possibly used for ECM identification without additional immunofluorescent staining of MSCs.

Interestingly, ECM is located not in the same direction as underlying MSCs and the components in ECM do not show any specific orientation (Fig. 3e–h). These results conform with recently published study which demonstrated the increase of bovine bone marrow MSC ECM production, but the lack of fiber organization capabilities when comparing to meniscal fibrochondrocytes. The researchers showed that co-culture can be used as a technique of balancing the synthetic properties of MSCs and the matrix remodeling capabilities of other cell types for tissue engineering applications [61]. It is known that MSCs can remodel their environment by simultaneous degradation of the scaffolds and deposition of newly synthesized ECM [62]. Our study shows that QDs have a huge potential in tissue engineering as nonspecific dye for staining dynamic ECM of viable human bone marrow MSCs.

To our knowledge, Auletta et al. were the first to label human bone marrow MSCs with Qtracker 625 QDs. The researchers investigated the biodistribution and mechanism of action of these cells on graft-versus-host disease and graft-versus-leukemia activity following bone marrow transplantation in mice model. By using novel microscopic cryo-imaging they were the first to show that human MSCs migrate to the marginal zone of the spleen and regulate donor T cell proliferation preserving allo-specific graft-versus-leukemia response [63]. These results highlight the feasibility of tracking QDs-labeled MSCs in vivo and open opportunities for mechanism of action investigations in other disease models.

Previously human MSCs were labeled with various QDs, including QDs525, QDs565, QDs585, QDs605, QDs655, and QDs800 [64, 65]. In our study we for the first time showed that QDs label human bone marrow MSC body, cell cytoplasmic protrusions and secreted ECM, thus revealing a more detailed MSC culture picture. QD size and charge are the key factors influencing QDs loading [66]. All this, including MSC tissue of origin, could explain the exclusive localization of QDs both inside and outside human bone marrow MSCs in vitro. Additionally, we for the first time used FLIM method to observe QDs localization in/on the extracellular matrix and showed that by analyzing mean fluorescence lifetime of QDs it is possible to identify and distinguish localization of QDs in various extracellular and intracellular structures without additional staining assays.

Recently we have demonstrated that QDs behave similarly in skin MSCs [25] indicating the potential universality of MSC staining method we are applying. However, several authors concluded that the characteristics of MSCs are tissue source-dependent. Al-Nbaheen et al. showed that human skin MSCs and human bone marrow MSCs exhibit differences in their proliferation, differentiation and molecular phenotype, which should be taken into consideration when planning their use in clinical protocols [67]. Liu et al. showed that human bone marrow MSCs and human skin MSCs differ in growth characteristics, gene expression and cytokine secretion profiles [68]. Reinisch et al. showed that human bone marrow, but not skin MSCs spontaneously formed hematopoietic niche in vivo [69]. Therefore, it might be too early to claim that all MSCs irrespective of origin would behave the same and the more precise experiments are required additionally for sufficient and conclusive evaluation of results.

## Conclusions

In this study we successfully labeled human bone marrow MSCs with QDs without delivering vehicles. Human bone marrow MSC culture density is one of the key factors determining QDs localization: in more dense culture QDs preferably label MSC extraordinary filopodia-like and extracellular structures. To our best knowledge, we for the first time labeled MSC extracellular matrix with non-targeted carboxylated quantum dots and showed FLIM advantages for distinguishing QDs, allowing calculation of their mean photoluminescence lifetime and identifying their localization in/on intracellular and extracellular structures without additional staining techniques. These results are promising in fundamental stem cell biology as well as cellular therapy, anticancer drug delivery and provide a basis for further tissue engineering investigations.

## Methods

### Study design

This study was performed in parallel with the study of mesenchymal stem cell (MSC) genetic characteristics published previously [31]. MSCs were isolated from bone marrow of 3 healthy donors. All donors have signed an Informed patient consent approved by the Vilnius Regional Committee of Biomedical Research (Lithuania, 2011-09-06 permission No. 158200-09-381-104). Isolated MSCs were expanded in vitro, characterized as proposed by The International Society for Cellular Therapy [31] and labeled with quantum dots (QDs). In this study, the QDs uptake dynamics, cytotoxity and biodistribution at subcellular level were investigated. The experiments were performed in the way to meet all three criteria for genuine replication [70]: (1) different donor cells randomized to flasks/wells independently, (2) different donor cells treated independently and no spillover, (3) different donor cells cultivated independently, thus, were not influencing each other.

### MSCs extraction using red blood cell lysis

MSCs were isolated from 6 ml (1 vacutainer) of bone marrow aspirate using red blood cell lysis method as previously described [34]. Briefly, all volume of vacutainer (6 ml) was transferred to the 50 ml conical centrifugation tube (BD Biosciences, France) and Erythrocyte lysis buffer (Qiagen GmbH, Germany) was added in the proportion 1:5. The tube was mixed for 1 min and centrifuged at 480×*g* for 5 min. After centrifugation the top layer was discarded and the pellet was resuspended with 5 mL of RPMI 1640 medium (Gibco, Great Britain) and washed twice using centrifugation. All cells were seeded into 75 cm^2^ ventilated flask and cultivated for 24 h in the Dulbecco’s Modified Eagle Medium (Lonza, Belgium) containing 10% of fetal bovine serum (FBS) (Invitrogen, USA) at 37 °C under a humidified 5% CO_2_ atmosphere allowing the cells to adhere to the culture flask.

### MSCs cultivation

Non-adherent cells were removed after 24 h by washing with phosphate buffered saline (PBS) solution (Gibco, USA). Human MSC basal medium (StemCell Technologies Inc., Canada) containing 10% of FBS for human MSCs (StemCell technologies Inc., Canada) was used for subsequent cultivation of MSCs. The medium was changed every 3–4 days. When adherent cells became subconfluent, MSCs were treated with trypsin–EDTA (Gibco, USA), washed twice with PBS, calculated and seeded in the new 75 cm^2^ (BD Biosciences, France) flasks under the density of 4000 cells per cm^2^. The cells were incubated in a humidified 5% CO_2_ incubator at 37 °C. All procedures were performed in the class II vertical laminar safety cabinet (Kojair, Singapore). MSCs from all donors were subcultured and investigated at passage 3.

### MSCs staining with Oil Red O

Samples were stained with 0.5% Oil Red O stain dissolved in isopropanol. Before the procedure Oil Red O solution was mixed with PBS in proportions 3:2 and then filtered with a sterile polyvinylidene Rotilabo^®^-syringe filters (Carl Roth GmbH + Co. KG, Germany) with 0.22 µm pore size.

### Labeling MSCs with quantum dots

MSCs were labeled using Qdot^®^ 625 ITK™ Carboxyl quantum dots (QDs) with a photoluminescence (PL) peak at 625 nm (Invitrogen, USA). They are amphiphilic polymer coated CdSe/ZnS QDs with carboxyl groups, average hydrodynamic diameter of 14.2 nm and zeta potential − 32.97 mV. A layer covering QDs allows facile dispersion of the quantum dots in aqueous solutions with retention of their optical properties [71]. For more physicochemical characteristics of QDs, view supplementary information (Additional file 5). To evaluate QDs uptake dynamics, intracellular and extracellular localization, MSCs were harvested at P2 and seeded at a density of 5000 cells/cm^2^ and 20,000 cells/cm^2^ (for extracellular localization evaluation) in 8-well chambered cover-slips (Nunc, USA) for confocal fluorescence microscopy and allowed to grow for 1 day. Then MSCs were incubated in full serum media with QDs (8 nM) over a time course ranging from 15 min to 24 h (37 °C, 5% CO_2_).

### Analysis of QDs uptake and viability of QDs-labeled MSCs

For quantitative analysis of QDs uptake, MSCs were seeded at a density of 20,000 cells/cm^2^ in 12-well plates (TPP, Switzerland) and allowed to grow for 2–3 days. Then MSCs were incubated with QDs (8 nM) over a time course ranging from 1 to 24 h (37 °C, 5% CO_2_). Flow cytometric analysis was carried out with a FACSort (BD Biosciences, USA). The data were analyzed with FlowJo (Tree Star, Ashland, OR) software. A minimum of 10 000 viable cells were measured per sample. Using forward and side scatter profiles and propidium iodide staining, debris and dead cells were gated out, respectively. Viability was calculated as a percentage of viable cells per sample. The results were presented as mean ± SD from three independent experiments.

### Imaging of QDs distribution in MSC culture

After indicated time of incubation, cells were routinely rinsed 3 times with pre-warmed human MSC basal medium (StemCell Technologies Inc., Canada) containing 10% of FBS for human MSCs (StemCell technologies Inc., Canada) and then were analyzed using a confocal laser scanning microscope (Nikon Eclipse TE2000-S, C1 plus, Nikon, Tokyo, Japan) equipped with CO_2_ Microscope Stage Incubation System (OkoLab, Italy). Additionally, DIC and phase contrast microscopy were used to visualize the morphological characteristics of MSC treated with QDs. A diode laser for 405 nm and an argon laser for 488 nm excitation coupled with a 60× NA 1.4 oil immersion objective (Plan Apo VC, Nikon, Japan) were used for all measurements. To detect Hoechst (Sigma Aldrich, USA) fluorescence emission (*λ*_ex_ = 405 nm) the 450/35 nm band pass filter was used. Fluorescence of Alexa-Fluor 488 nm-conjugated transferrin (Invitrogen, USA), Alexa-Fluor 488 nm-conjugated phalloidin Invitrogen, USA) was detected using a 515/30 band pass filter (*λ*_ex_ = 488 nm) as well as fluorescence of mouse anti-human CD44 antibody conjugated with Alexa-Fluor 488 (Thermo Fisher Scientific, USA) and fibronectin (Gibco, Thermo Fisher Scientific, USA). To visualize QDs fluorescence (*λ*_ex_ = 488 nm) the 605/75 nm band pass filter was applied. Laser scanning was controlled by the Nikon EZ-C1 software; individual color channels were recorded separately to minimize spectrum overlap. The images were further processed using the EZ-C1 Bronze version 3.80 (Nikon, Japan) and ImageJ 1.41 software (NIH, USA).

### Fluorescence-lifetime imaging microscopy (FLIM) analysis

FLIM images were obtained using Lifetime and FCS Upgrade for Nikon C1si (PicoQuant GmbH, Berlin, Germany). The imaging system was composed of a pulsed diode laser (405 nm) with a pulse width of 39 ps and a repetition rate of 10 MHz. Detected photons were counted by a time correlated single-photon counter PicoHarp 300 (PicoQuant GmbH, Berlin, Germany). Excited states’ PL lifetime signal of QDs in MSCs was investigated with a single channel unit of single photon-counting avalanche photodiodes (SPAD) at a spectral range of 650 ± 75 nm. Each one of FLIM images was acquired by collecting 1000 counts at the peak value. The image resolution was fixed at 512 × 512 pixels and images reconstruction work was performed using a three-exponential fitting model (SymPhoTime software, PicoQuant GmbH, Germany).

## Additional files

**Additional file 1.** Movie showing living mesenchymal stem cells under effect of trypsin after incubation with QDs for 24 h.

**Additional file 2.** Living mesenchymal stem cells movie showing staining of extracellular matrix and intracellular accumulation of QDs. ROI 1 and 2 marking areas with stained ECM.

**Additional file 3.** 3D (z-stack) reconstruction movie showing MSCs after incubation with QDs for 6 h.

**Additional file 4.** 3D (z-stack) reconstruction movie showing MSCs after incubation with QDs for 24 h.

**Additional file 5.** Characterization of carboxylated QDs. (**a**) Image of QDs was made by Philips CM200 Field emission transmission electron microscope (TEM) equipped with light element EDX detector and Gatan Imaging Filter for PEELS and Energy Filtered TEM. Scale bar 20 nm. (**b**) 3D topography image of QDs was registered on mica surface with Innova atomic force microscope in the tapping mode using silicon nitride probes MPP12283. (**c**) Hydrodynamic size distribution of QDs was measured using a dynamic light scattering device Zeta Plus PALS. (**d**) The steady state absorption and photoluminescence spectra were recorded (*λ*_ex_ = 625 nm) on Cary 50 UV–Vis spectrophotometer and Cary Eclipse fluorimeter, respectively. (**e**) PL decay curve of QDs was registered using FLS920 spectrometer equipped with 405 nm (66.9 ps) pulsed laser.

## Abbreviations

DIC: differential interference contrast
ECM: extracellular matrix
FBS: fetal bovine serum
FLIM: fluorescence-lifetime imaging microscopy
MRI: magnetic resonance imaging
MSCs: mesenchymal stem cells
PBS: phosphate buffered saline
PL: photoluminescence
QDs: quantum dots
ROI: region of interest
SD: standard deviation
SPIO: superparamagnetic iron oxide
TEM: transmission electron microscope
