# Supplementary figures and images for: Uptake and distribution of carboxylated quantum dots in human mesenchymal stem cells: cell growing density matters
Source: J Nanobiotechnology. 2019 Mar 13;17:39. doi: 10.1186/s12951-019-0470-6 (PMC6417192; doi:10.1186/s12951-019-0470-6)

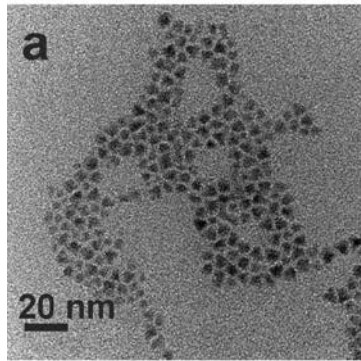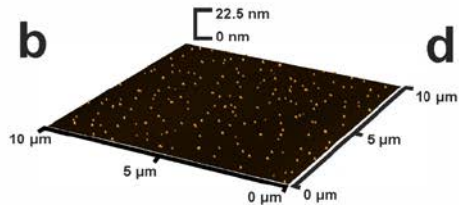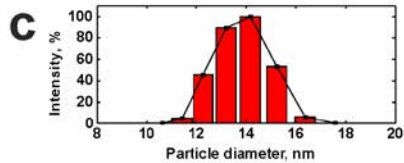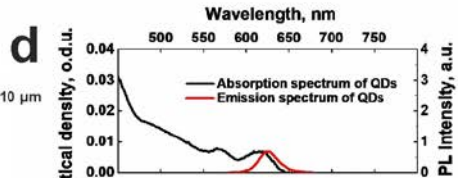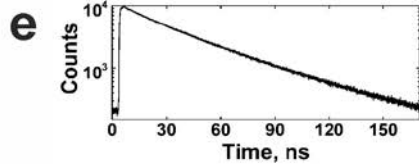

Supplement: Supplementary file 5 — Additional file 5. Characterization of carboxylated QDs. (a) Image of QDs was made by Philips CM200 Field emission transmission electron microscope (TEM) equipped with light element EDX detector and Gatan Imaging Filter for PEELS and Energy Filtered TEM. Scale bar 20 nm. (b) 3D topography image of QDs was registered on mica surface with Innova atomic force microscope in the tapping mode using silicon nitride probes MPP12283. (c) Hydrodynamic size distribution of QDs was measured using a dynamic light scattering device Zeta Plus PALS. (d) The steady state absorption and photoluminescence spectra were recorded (λex = 625 nm) on Cary 50 UV–Vis spectrophotometer and Cary Eclipse fluorimeter, respectively. (e) PL decay curve of QDs was registered using FLS920 spectrometer equipped with 405 nm (66.9 ps) pulsed laser. [file 12951_2019_470_MOESM5_ESM.pdf]
